# The acoustically evoked short latency negative response (ASNR) in a unilaterally deaf cat with histologically-confirmed cochleosaccular degeneration

**DOI:** 10.1186/s12917-020-02426-z

**Published:** 2020-06-26

**Authors:** Ezio Bianchi, Anna Maria Cantoni, Luc Poncelet

**Affiliations:** 1 Department of Veterinary Science, University of Parma, Via del Taglio, 10, 43126 Parma, Italy; 2 Laboratory of Anatomy, Biomechanics and Organogenesis, CP 619, Faculty of Medicine, Free University of Brussels, route de Lennik, 808, B-1070 Bruxelles, Belgium; 3 ULB Neuroscience Institute, Bruxelles, Belgium

**Keywords:** Cat, Cochleosaccular degeneration, Deafness, Acoustically evoked short latency negative response, Vestibular evoked myogenic potentials

## Abstract

**Background:**

A negative potential is occasionally recorded in humans and animals with profound deafness during brainstem auditory evoked potential (BAER) tests if loud intensities are used. This acoustically evoked short latency negative response (ASNR) is hypothesized to be of saccular origin. The sensitivity to sound of vestibular end organs is also used to produce vestibular evoked myogenic potentials (VEMP), a test that evaluates vestibular function. The same saccular origin is accepted also for VEMP.

**Case presentation:**

A neutered male white domestic short hair cat presented with profound deafness and an ASNR in the left ear during BAER test performed when he was 8 months old. BAER tracings were substantially unchanged at the age of 12 years, immediately before euthanasia that was requested by the owner for the presence of an unrelated neoplastic disorder. The cat underwent a complete post-mortem necropsy including histopathology of the middle and inner ears. Histopathologic results confirmed the presence of a cochleosaccular degeneration of the left ear while the cochlea and sacculus of the right ear and the utriculus and semicircular canals of both ears were histologically normal.

**Conclusions:**

This case report describes the auditory and histopathologic findings of a cat that showed an ASNR during BAER test despite the presence of cochleosaccular deafness. These results confirm that a saccular origin for the ASNR in this case, and in general in cats and dogs with congenital deafness associated with white pigmentation, is improbable. The hypothesis that the sacculus is the vestibular end organ responsible for the generation of the ASNR and VEMP in humans comes mainly from animal studies. The findings in this report may change the clinical interpretation of the results of BAER and VEMP not only in companion animals, but in humans as well.

## Background

Sensitivity of vestibular receptors to loud sounds has been demonstrated in mammals [1, 2]. Brainstem auditory evoked potentials (BAER) testing using loud stimuli show a vertex-negative potential with a latency of 3 ms in some human patients with profound deafness of cochlear origin. This deflection has been termed N3 potential or acoustically evoked short latency negative response (ASNR). The ASNR has also been reported in dogs and cats with congenital sensorineural deafness associated with a white coat [3]. Approximately 25 years ago, researchers started to take advantage of the acoustic sensitivity of vestibular receptors to produce vestibular evoked myogenic potentials (VEMP), a short-latency vestibular response recorded from neck muscles in response to intense sound or vibration. VEMP are commonly used as a clinical tool for objective evaluation of vestibular function [4]. The ASNR and VEMP are thought to be the result of activation of the sacculus [4–6]. The presence of the ASNR in dogs and cats with congenital profound deafness associated with white pigmentation is in contrast with a saccular origin of these potentials because these animals often have cochleosaccular degeneration [7–9]. In this paper we describe the clinical, audiometric and histopathologic findings of the middle and inner ear of a white cat with unilateral cochleosaccular deafness and the presence of an ASNR. We also discuss the possible sense organ and generator involved in the ASNR and VEMP generation.

## Case presentation

**Fig. 1:**
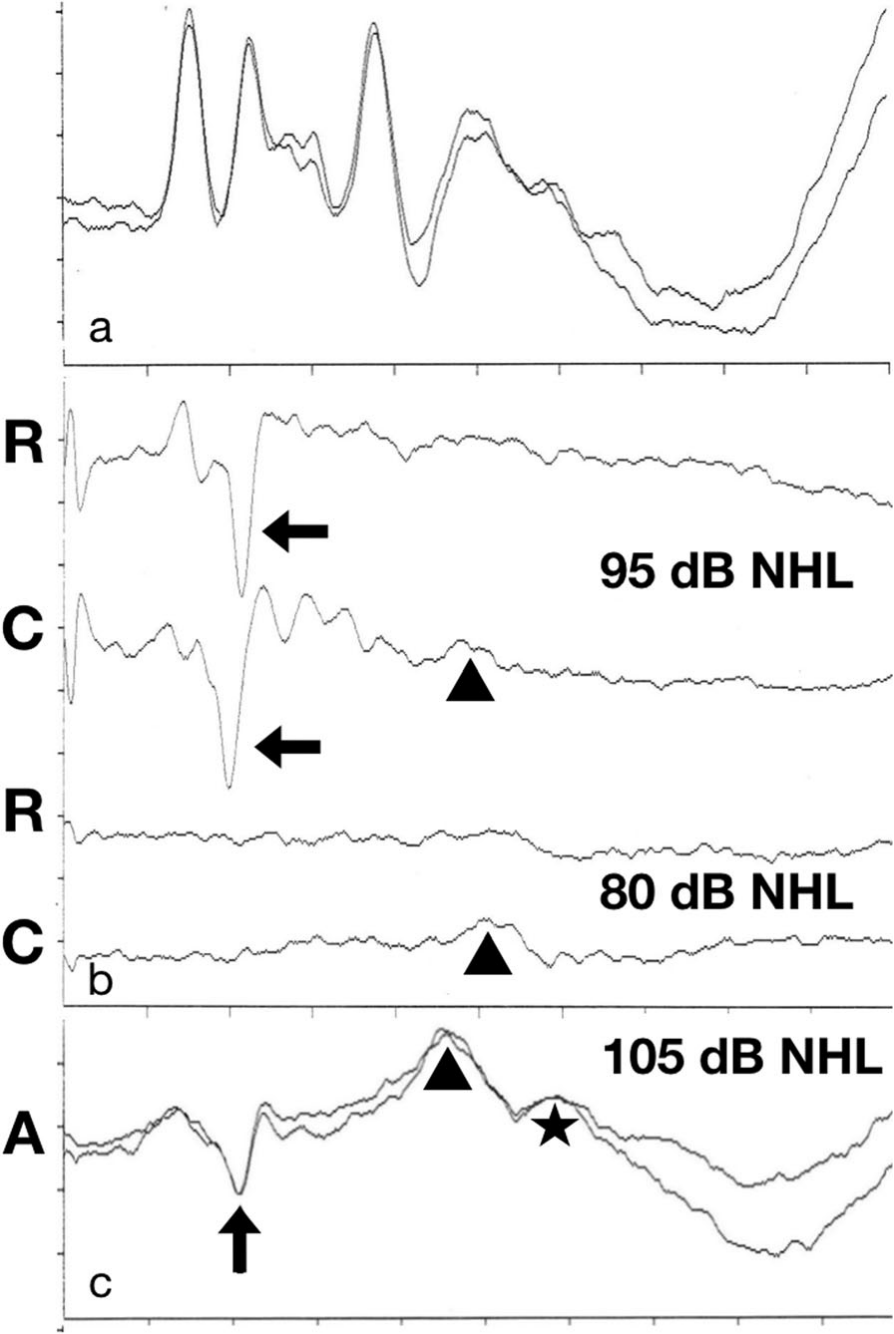
BAER tracings. **a**: right ear, normal waveform recorded at the age of 12 years using alternating stimulus polarity and an intensity of 90 dB NHL. **b**: left ear, waveforms recorded at the age of 8 months using rarefaction (R) and condensation (C) stimulus polarity. **c**: left ear, waveforms recorded at the age of 12 years using alternating (A) stimulus polarity. Using high stimulus intensities a V-shaped negative potential with a latency of approximately 2 ms (ASNR; arrows) is recorded in the left ear. The ASNR is not recorded in the same ear at 80 dB NHL. The ASNR is affected by click polarity but without phase reversal. Peaks V (arrowheads) and VI (star) are produced in the left ear by unwanted stimulation of the contralateral ear (crossover effect). Vertex-ipsilateral mastoid montage. **a, c**: 1 ms/Div; 0.31 μV/Div. **b**: 1 ms/Div; 0.62 μV/Div

A neutered male white domestic short hair cat underwent BAER test at 8 months of age that revealed profound deafness of the left ear and the presence of the ASNR. BAER testing of both ears was performed using an electrodiagnostic equipment (Neuropack Four Mini MEB 5304 K – Nihon Koden – Japan) with the methodology described elsewhere (Bianchi et al., 2006). Briefly, the signal was amplified 200,000 times, filtered with a bandwidth of 100–3000 Hz, and averaged 500 times. Automatic artefact rejection was used with an analysis time of 10 ms. Two recording channels were used. For the first channel the montage was the following: vertex (non-inverting input of the amplifier) and ipsilateral mastoid (inverting input). For the second channel the montage was: vertex (non-inverting input of the amplifier) and second cervical vertebra (nape) (inverting input). Ground electrode was inserted at the base of the neck. Recording electrodes and ground electrode were stainless steel needles. Rarefaction, condensation and alternating clicks produced by electrical square waves of 0.1 ms were presented with a delivery rate of 10/s using an insert earphone (0.30 ms delay) at intensities of 80, 90, 95 and 105 dB NHL. For each stimulus intensity, two tracings were obtained and superimposed to show reproducibility of the responses. A masking noise of 40 dB below the click intensity was delivered to the untested ear. A normal BAER tracing was recorded in the right ear using a stimulus intensity of 90 dB NHL (Fig. 1a). A V-shaped negative potential with 2 ms latency was observed in the tracings of the left ear when the acoustic stimulus intensity was increased to 95 dB NHL and over (Fig. 1b). As previously reported, in the present case the latency and morphology of ASNR was mildly affected by stimulus polarity but without phase reversal [3]. The effect of click polarity on ASNR contrasted with that observed on cochlear microphonic potentials.

BAER test was repeated on the cat at the age of 12 years, immediately before euthanasia that was performed at the owner’s request for the presence of an unrelated neoplastic disorder. In this case the BAER test protocol was limited to the use of alternating clicks with the aim of confirming the presence of the ASNR. In both ears the recorded tracings were similar to those recorded previously. The threshold of ASNR was increased by approximately 10 dB (Fig. 1c). Despite the use of contralateral masking, some crossover effect was present when stimulating the left ear (Fig.1b, c).

The cat underwent a complete post-mortem necropsy including histopathology of middle and inner ears.

Temporal bones were removed immediately after euthanasia, bullae were opened and the samples were immersed in 10% formalin. The samples were decalcified in 5% formic acid for 4 weeks with weekly changes. They were embedded in paraffin and sectioned at 5 μm. Rehydrated sections were stained with haematoxylin and eosin.

**Fig. 2:**
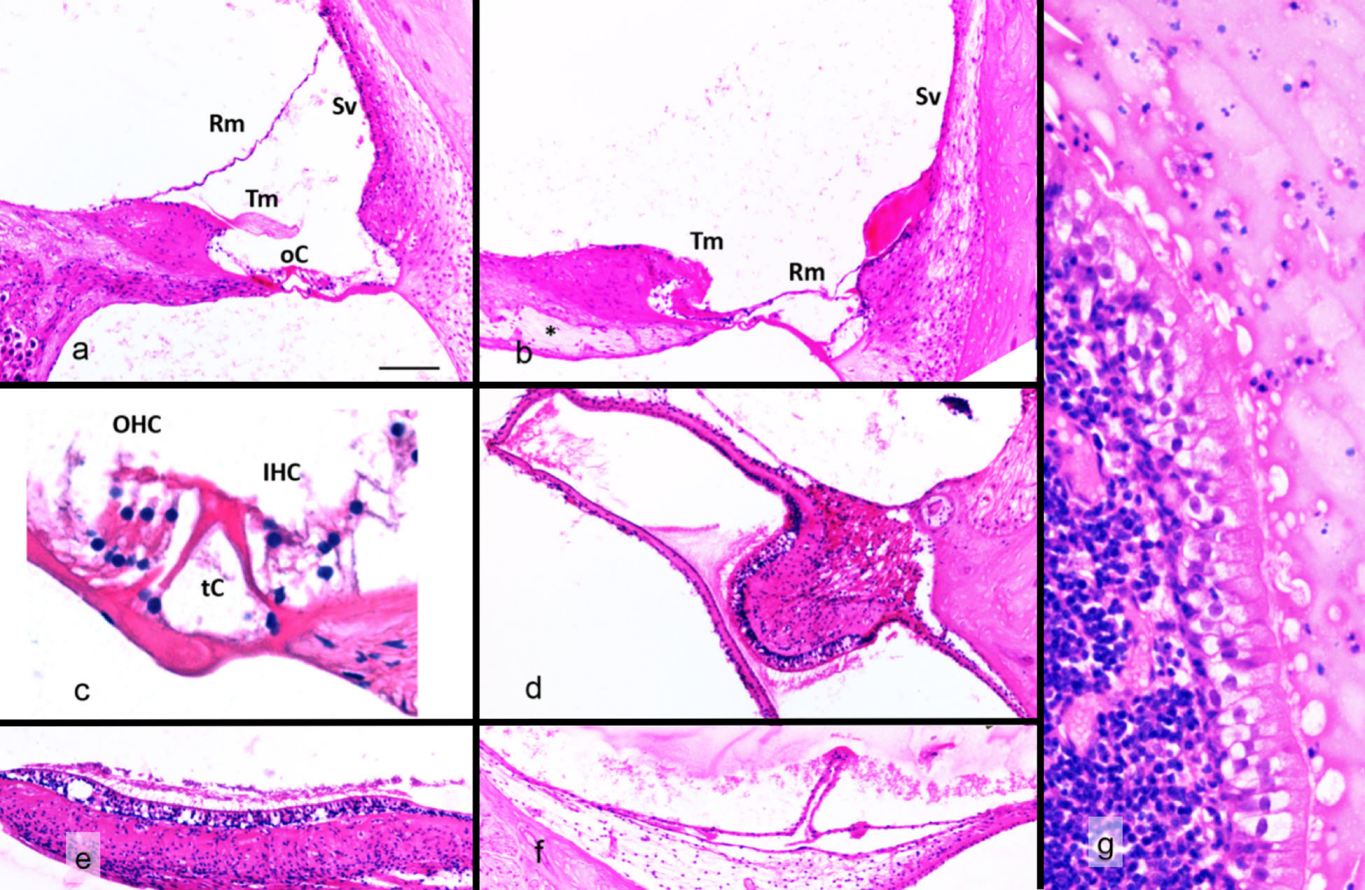
Inner ears features. **a**: cochlea, right ear; **b**: cochlea, left ear. Rm: Reissner membrane; SV: stria vascularis; Tm: tectorial membrane; oC: Organ of Corti; *: Spiral ganglion. **c**: Organ of Corti, right ear. OHC: outer hair cells; IHC: inner hair cells; tC: tunnel of Corti. **d**: intact semi-circular ampulla in the left ear. **e**: intact utricular macula in the left ear. **f**: collapsed sacculus with no sensory epithelium recognizable, left ear. **g**: both middle ear presented chronic inflammation (mononuclear cell accumulation covered by a respiratory type epithelium, with some exudate including polymorphonuclear cells). Bar = 100 μm in **a**, **b**, **d**, **e**, **f** and 25 μm in **c** and **g**. Haematoxylin and eosin

The right ear was normal, except for some fixation artefacts. The scala media, limited by the basilar and Reissner membranes, was intact. The stria vascularis displayed its normal three - cell layer structure. The organ of Corti with inner and outer hair cells, pillar and Deiter’s cells, Nuel’s spaces and Corti’s tunnel were perfectly recognizable, as well as a normally shaped tectorial membrane (Fig. 2a, b). The left inner ear exhibited the typical changes of cochleosaccular degeneration: the stria vascularis was reduced to a single layer of flat cells, the Reissner’s membrane was collapsed, the tectorial membrane was rolled into the inner sulcus, and there was no recognizable organ of Corti (Fig. 2c). In addition, the sacculus was collapsed with no visible macula (Fig. 2d). Furthermore, the semi-circular canal ampullae were intact with normally shaped cristae and the utriculus included a normally shaped macula (Fig. 2e, f). Descriptions of inner ear changes in cochleosaccular deafness are usually described in young animals and do not show changes in the spiral ganglion. In the present case, in this older animal, all the spiral ganglion neurons had disappeared (Fig. 2c).

Both middle ears presented chronic inflammation characterized by mononuclear cell accumulation covered by a respiratory type epithelium, with some exudate including polymorphonuclear cells in the bulla (Fig. 2g). However, inflammatory cells were not found in the inner ears.

## Discussion and conclusions

This case report describes the auditory and histopathologic findings of a cat that showed an ASNR during BAER test despite the presence of a cochleosaccular deafness. The negative potential recorded in the left ear was reproducible in the same ear over time, while the BAER tracing of the right ear remained normal. The peculiar V-shape, the latency, and the dependence on high stimulus intensities makes this vertex-recorded prominent negative peak strikingly similar to the ASNR recorded in some profoundly deaf patients [5, 6] and in dogs and cats with cochleosaccular deafness [3]. Responses to increasing stimulus frequency and to stimulus polarity, as well as the latency (approximately 2 ms) indicate a neural potential and not a receptor potential and vestibular nuclei might be the best candidates for ASNR generation [3]. A contribution by the contralateral ear to the generation of ASNR cannot be completely excluded in this case considering the presence of some crossover effect during BAER testing. The level of the contralateral masking noise (− 40 dB) was lower than that used in other studies (− 30 dB). Nevertheless, the waveform morphology and the reported presence of ASNR also in animals with bilateral profound deafness is suggestive of an ipsilateral origin.

Cochleosaccular deafness is generally associated with a white coat and is due to a primary degeneration of the endolymph-producing stria vascularis, seemingly as a consequence of the absence of its intermediate cells, which are specialized melanocytes [10]. Utriculus and semicircular canals are intact in these animals because these structures rely on the dark cell area integrity for endolymph production [11].

Cochleosaccular degeneration was confirmed in this cat based on the histopathological results that showed a degeneration of the sacculus and cochlea in the left ear. Therefore, we can assume that the ASNR did not originate from the sacculus. As in this specific case, it may also be true in general for dogs and cats with white mantle- associated cochleosaccular deafness. Utriculus and semicircular canal receptors, that were intact also in the left ear, represent the main candidates for ASNR origin in this type of deafness.

The true relative contribution of the different vestibular end organs to sound sensitivity implicated in the generation of the ASNR and VEMP is still an object of discussion. Although some studies evidenced loud sound sensitivity from all vestibular receptors [1, 12, 13], other papers identified the sacculus as the main sound sensitive vestibular organ [14, 15]. On the basis of these animal studies, the sacculus is commonly accepted as the click sensitive vestibular organ implicated in the generation of the ASNR and VEMP.

Based on the results of our case report, ASNR is also present in animals with non-functional sacculi. Therefore, utriculus and semicircular canal receptors may have, at least partially, a role in the generation of the ASNR and possibly also VEMP. These findings have an impact on the interpretation of ASNR and VEMP in humans and animals. Further studies aimed at clarifying the precise contribution of the different vestibular end organs to the acoustically evoked vestibular responses are needed.

## Data Availability

All data generated or analysed during this study are included in this published article.

## Abbreviations

ASNR: acoustically evoked short latency negative response
VEMP: vestibular-evoked myogenic potentials
BAER: brainstem auditory evoked potentials
